# Extracellular Vesicles from Osteotropic Triple-Negative Breast Cancer Cells Transfer miRNAs to Bone Cells Reducing Collagen Expression and Bone Matrix Mineralisation

**DOI:** 10.3390/pharmaceutics18030317

**Published:** 2026-03-02

**Authors:** Luca Giacchi, Argia Ucci, Elisa Pucci, Loreto Lancia, Fanny Pulcini, Simona Delle Monache, Nadia Rucci, Marco Ponzetti

**Affiliations:** 1 Department of Biotechnological and Applied Clinical Sciences, University of L’Aquila, 67100 L’Aquila, Italy; luca.giacchi@graduate.univaq.it (L.G.); argia.ucci@univaq.it (A.U.); elisa.pucci@graduate.univaq.it (E.P.); loreto.lancia1@graduate.univaq.it (L.L.); fanny.pulcini@univaq.it (F.P.); simona.dellemonache@univaq.it (S.D.M.); mp.univaq@gmail.com (M.P.); 2 Department of Rare Endocrine Disorders, Novo Nordisk A/S, 2760 Maaloev, Denmark

**Keywords:** breast cancer, miRNAs, bone metastases, osteoclast, osteoblast, collagen

## Abstract

**Background/Objectives**: Bone metastases are a common complication of breast cancer. In our previous study, we reported that extracellular vesicles released by osteotropic human (MDA-MB-231) and murine (4T1) breast cancer cells disrupt bone homeostasis by enhancing osteoclast differentiation and impairing osteoblast function. Based on these findings, we investigated whether microRNAs contained within tumour-derived EVs could mediate these bone-altering effects. **Methods**: MDA-MB-231- and 4T1-EVs were tagged with the RNA-specific fluorophore SYTORNA and employed to treat mouse primary bone marrow macrophages (BMMs) and osteoblasts (OBs). We also performed RNAseq on MDA-MB-231- and 4T1-EVs to assess their miRNAs content. Finally, we evaluated the effect of selected miRNA-mimics on OBs, BMMs and HUVEC cells. **Results**: Fluorescence microscopy demonstrated EV-RNAs shuttling to recipient cells, while RNA sequencing on MDA-MB-231- and 4T1-EVs revealed that, of the top 20 expressed miRNAs, 10 were common. Among them, we first focused on the following four: miR-26a-5p, miR-24-3p, miR-29a-3p, and miR-29b-3p, which were linked to bone biology. We confirmed their presence in MDA-MB-231-/4T1-EVs by qPCR. Then, we evaluated their EV-mediated shuttling to BMMs and OBs using affinity tags. Among all the conditions tested, miR-29a and miR-29b were the best-shuttled miRNAs, with efficiency between 50–100% in both OBs and BMMs, both for MDA-MB-231- and 4T1-EVs. Finally, to test whether miR-29a and miR-29b could have a functional role in bone cells, OBs were transfected with miR-29a and 29b-mimics, discovering that this treatment reduced collagen1α1 and 1α2 mRNA as well as the OBs’ mineralisation ability, while the same miRNA mimics were found to have no effect on osteoclastogenesis or on in vitro angiogenesis. **Conclusions**: MDA-MB-231- and 4T1-EVs shuttle miRNAs to bone cells, which likely contributes to OBs’ activity impairment.

## 1. Introduction

Extracellular vesicles (EVs) are lipid bilayer nanoparticles, 50–1000 nm in diameter, used by virtually every cell type in the body to shuttle multi-molecular messages [1]. MicroRNAs are abundant in EVs, especially in the subpopulation termed “small EVs” (sEVs, 50–150 nm diameter), including the ones arising from breast cancer (BrCa) cells [2,3]. Cancer EV-miRNAs are involved in reprogramming the host microenvironment, to increase cancer growth or metastatic spreading [4,5]. Bone is the preferential site of metastasis in the most frequently occurring human neoplasias, including BrCa, and the involvement of EV-shuttled miRNAs in BrCa bone metastasis is still a topic deserving more investigation [6,7,8]. It is well established that tumour cells, either directly or through extracellular vesicles (EVs), can alter bone cells’ physiology to support their growth. Consistent with this concept, we previously demonstrated that BrCa-derived EVs disrupt bone homeostasis by impairing osteoblast differentiation, enhancing osteoclastogenesis, and promoting angiogenic responses [9]. These findings prompted us to investigate the molecular mediators underlying these effects. Since miRNAs are known to be the most abundant components in EVs, we hypothesised that specific microRNAs packaged and shuttled by BrCa-derived EVs could be responsible for mediating the observed anti-osteoblastogenic, pro-osteoclastogenic, and pro-angiogenic effects on the bone microenvironment. To test this hypothesis, we characterised the miRNome of BrCa-EVs and implemented an affinity-tag-based strategy to directly track and isolate EV-shuttled miRNAs in recipient bone cells. Moreover, we demonstrated that two of the shuttled miRNAs, miR-29a-3p and miR-29b-3p, could partially mediate the EVs’ effect by impairing osteoblast mineralisation ability and collagen production, but do not appear to influence osteoclastogenesis or angiogenesis in a cell-autonomous manner.

## 2. Materials and Methods

### 2.1. Materials

Dulbecco’s modified Minimum Essential Medium (DMEM, cat #ECB7501L), Dulbecco’s Phosphate Buffer Saline (DPBS, cat #ECB4004L), Foetal Bovine Serum (FBS, cat# 26140-079), penicillin/streptomycin (cat# ECB3001D), and L-glutamine (cat# ECB3000D) were from GIBCO (Uxbridge, UK) and Euroclone (Milan, Italy). Fifteen-well μ-slides (cat# 81506) were from Ibidi GmbH (Gräfelfing, Germany). Sterile plasticware was from Falcon Becton-Dickinson (Cowley, Oxford, UK) and Costar (Cambridge, MA, USA). The 3-(4,5-dimethyl-2-thiazolyl)-2,5-diphenyl-2H-tetrazolium bromide (MTT, cat #M2128), Histopaque 1077 (cat #10771-100ML), TRACP (cat #387A-1KT) activity detection kits, *Clostridium histolyticum* type IV collagenase (cat #C5138-1G) and the crystal violet dye were supplied by Sigma-Aldrich (St. Louis, MO, USA). The SYTO^®^ RNAselect (cat #S32703), TaqMan Advanced miRNA cDNA kit (cat #A28007), TaqMan Advanced miRNA Assay (cat #A25576, RackID #P03229606), TaqMan Fast Advanced Master Mix (cat #4444556), RevertAid First Strand cDNA Synthesis Kit (cat #K1622), Lipofectamine 3000 (cat #L3000-075) and the Click-iT™ Nascent RNA Capture Kit (cat #C10365), were purchased by Thermo Fisher Scientific (Waltham, MA, USA). The miRNeasy Micro Kit (cat #217084) was from Qiagen (Germantown, MD, USA). TRIzol (cat #15596018) was purchased by Thermo Fisher Scientific (Waltham, MA, USA), the Luna^®^ Universal qPCR Master Mix (cat #M3003L) was from New England BioLabs (Ipswich, MA, USA). The human recombinant macrophage stimulating factor (M-CSF, cat #300-25) and the human receptor activator of nuclear factor kappa-Β ligand (hRANKL, cat #310-01) were from PeproTech (Cranbury, NJ, USA). All other reagents were purchased by Sigma-Aldrich (St. Louis, MO, USA).

### 2.2. Cell Cultures

The human BrCa cell line MDA-MB-231 and the mouse BrCa cell line 4T1 were obtained by the American Tissue Culture Collection (ATCC, Rockville, MD, USA) and grown in DMEM supplemented with 10% FBS, 100 IU/mL penicillin, 100 μg/mL streptomycin, and 2 mM L-glutamine. The human umbilical vein endothelial cells (HUVECs) were purchased by Lonza (Basel, Switzerland) and cultured in endothelial cell growth medium (EGM)-2. All cells were grown in a humidified 95% air/5% CO_2_ incubator at 37 °C.

### 2.3. Extracellular Vesicles Isolation

Extracellular vesicles (EVs) were purified following previously established protocols [9,10] with minor procedural adjustments. MDA-MB-231 and 4T1 cells were grown until 70–80% confluence, then washed with DPBS and incubated in serum-free DMEM for 24 h to avoid contamination from serum-derived vesicles. Conditioned media (CM) were collected and subjected to a series of differential centrifugation steps to remove cells and debris. Samples were first centrifuged at 300 *g* for 5 min at 4 °C to eliminate residual cells, followed by centrifugation at 5000 *g* for 25 min at 4 °C to discard larger cellular fragments. The resulting supernatant was then transferred to ultracentrifuge tubes and processed in a Beckman L7-65 ultracentrifuge equipped with an SW41-Ti rotor (Beckman Coulter, Indianapolis, IN, USA). Ultracentrifugation was performed at 100,000 *g* for 70 min at 9 °C to pellet extracellular vesicles. After removal of the supernatant, EV pellets were gently resuspended in DPBS or in the appropriate experimental buffer, depending on downstream applications (Section 2.4, Section 2.5, Section 2.6, Section 2.7, Section 2.8, Section 2.9, Section 2.10 and Section 2.11).

### 2.4. Nanoparticles’ Tracking Analysis

Extracellular vesicles were obtained from 12 mL of CM from MDA-MB-231 and 4T1 cultures and subsequently resuspended in 100 μL of 0.22 μm–filtered DPBS. For particle size and concentration analysis, EV suspensions were diluted 1:100 in DPBS and analysed using a NanoSight NS300 instrument (Malvern Instruments, Malvern, UK). Instrument settings, including camera level and flow rate, were optimized according to the manufacturer’s recommendations and NS300 calibration guidelines. For each biological replicate, five independent 60-s recordings were acquired and averaged for analysis.

### 2.5. Transmission Electron Microscopy

For ultrastructural analysis, 10 μL of extracellular vesicle suspension (obtained from 12 mL of conditioned medium derived from MDA-MB-231 and 4T1 cells) was fixed with an equal volume of 2% glutaraldehyde for 30 min. The fixed samples were deposited onto Formvar/carbon-coated copper grids and left to adsorb for 20 min. Grids were subsequently rinsed with distilled water and negatively stained with 1% phosphotungstic acid (PTA) for 2 min. After additional washes in distilled water, grids were air-dried overnight at room temperature. Imaging was performed using a Philips CM100 transmission electron microscope (Amsterdam, The Netherlands) operating at 80 kV and equipped with a PHURONA digital camera (Emsis, Münster, Germany).

### 2.6. Western Blot Analysis

Extracellular vesicle pellets obtained from 12 mL of conditioned medium derived from MDA-MB-231 and 4T1 cells were lysed in RIPA buffer (50 mM Tris-HCl pH 7.5, 150 mM NaCl, 16.6 mM NP-40, 12.1 mM sodium deoxycholate, and 3.47 mM SDS) supplemented with protease inhibitors. Protein concentration was determined (BCA assay) and 15 μg of total EV protein was next separated by 15% SDS–polyacrylamide gel electrophoresis under reducing conditions. Proteins were transferred onto nitrocellulose membranes, which were subsequently blocked for 2 h at room temperature in 5% non-fat dry milk prepared in TBS-T. Membranes were then incubated with primary antibodies diluted in 1% milk either overnight at 4 °C or for 1 h at room temperature, depending on the antibody specifications. After washing, membranes were exposed to the appropriate HRP-conjugated secondary antibodies for 1 h at room temperature. Signal detection was carried out using SuperSignal chemiluminescent substrate, according to the manufacturer’s instructions, and images were acquired with a Bio-Rad Gel Doc XR+ system (Hercules, CA, USA). Densitometric analysis of band intensity was performed using ImageJ software 1.54p (NIH).

### 2.7. Evaluation of Extracellular Vesicle-Mediated RNA Transfer to Bone Cells

To visualize RNA transfer, EV-associated RNAs were labelled using SYTO^®^ RNASelect Green Fluorescent Cell Stain. Briefly, the dye was diluted 1:5 in DMSO and 1 µL of this solution was added to MDA-MB-231- and 4T1-EVs resuspended in PBS. Samples were incubated at 37 °C for 20 min to allow labelling of EV-associated RNA. Excess dye was removed by washing EVs with 33 mL of PBS and by ultracentrifugation at 100,000 *g* for 70 min at 8 °C. The final EV pellets were resuspended in PBS. Osteoblasts or BMMs were seeded on chamber slides at a density of 210,000 cells/cm^2^ and treated with either SYTO RNASelect-labelled, unlabelled EVs, or vehicle. RNA transfer was evaluated after 1, 6, and 12 h of incubation at 37 °C in a humidified atmosphere containing 5% CO_2_. Cells were then washed with PBS, fixed with 4% paraformaldehyde for 20 min at RT, and mounted using an anti-fade mounting medium containing DAPI. Uptake and intracellular localisation of EV-derived RNA were analysed by epifluorescence microscopy.

### 2.8. RNA Extraction

RNA extraction was performed using the miRNeasy Micro Kit (Qiagen), according to manufacturer’s guidelines. Briefly, cells were lysed to release total RNA, which was subsequently purified through selective binding to a silica membrane. After washing, RNA was eluted in 20 µL of RNase-free water. Extracted RNA was subjected to spectrophotometric quantification, prior to NGS and downstream click chemistry reactions.

### 2.9. Next-Generation Sequencing Analysis of Non-Coding RNA

RNA was extracted from MDA-MB-231- and 4T1-EVs using the miRNeasy kit. Purified RNA was then quantified and sent to a specialized sequencing facility (GenXPro GmbH, Frankfurt am Main, Germany) for downstream analysis. Sequencing was performed on all non-coding RNA species using Illumina chemistry (San Diego, CA, USA), with a sequencing coverage > 100×. Obtained read counts were normalised to the total number of reads (number of transcripts/million non-coding transcripts), allowing the identification and ranking of the 20 most abundant mature miRNAs present in EVs.

### 2.10. miRNA Transfer to Bone Cells

To evaluate the transfer of miRNAs from BrCa-EVs to bone cells, MDA-MB-231 and 4T1 cells were subjected to serum starvation in DMEM containing 0.1 mM 5-ethynyl uridine (5-EU) in order to label newly synthesized RNAs. After 24 h of incubation, CM was collected, and EVs were isolated, as previously described. The final EV pellets were resuspended in DMEM and used immediately for treatment of recipient bone cells. Treatments were carried out for 24 h at 37 °C in a humidified incubator with 5% CO_2_. RNA was extracted, as mentioned above, and subjected to a click chemistry-based reaction using the Click-iT™ Nascent RNA Capture Kit, following the manufacturer’s protocol, in order to selectively biotinylate 5-EU-labeled RNA molecules. RNA was purified using the miRNeasy Micro Kit and subsequently incubated with Dynabeads™ MyOne Streptavidin T1 in the presence of Click-iT RNA binding buffer. Samples were incubated at 70 °C for 5 min, followed by incubation at RT for 30 min under gentle agitation to allow binding of biotinylated RNA to the beads. Beads were then immobilised using a magnetic stand and sequentially washed with the appropriate Click-iT wash buffers to remove unbound RNA. Bead-bound RNA was then used for cDNA synthesis and real-time RT-PCR analysis. In addition, EVs derived from donor cells cultured without 5-EU were processed in parallel and served as negative controls to confirm specificity of labelling and enrichment.

### 2.11. TaqMan Analysis of miRNAs

MicroRNAs detection was performed by real-time RT-PCR using TaqMan Advanced miRNA assays, following the manufacturer’s protocol. All reactions were carried out using RNA enriched through the click chemistry-based procedure and bound to Dynabeads MyOne Streptavidin T1. Following resuspension of the beads in Click-iT Wash Buffer 2, samples were incubated at 70 °C for 5 min and directly subjected to the first step of the protocol, consisting of poly(A) tailing of mature miRNAs. Polyadenylation was performed in a thermal cycler at 37 °C for 45 min, followed by enzyme inactivation at 65 °C for 10 min. Next, ligation was carried out at 16 °C for 60 min. Reverse transcription was then carried out at 42 °C for 15 min, followed by a pre-amplification step (miR-Amp reaction). Real-time PCR analysis was performed using TaqMan Fast Advanced Master Mix and specific TaqMan Advanced miRNA assays. Reactions were run under the following cycling conditions: initial enzyme activation at 95 °C for 20 s, followed by 40 cycles of denaturation at 95 °C for 3 s and annealing/extension at 60 °C for 30 s. Fluorescence data were collected for both FAM and VIC fluorophores. Relative quantification was finally performed by analysis of cycle threshold (Ct) values. A difference of at least 2 Ct between treated samples and controls was considered indicative of successful microRNA transfer to recipient bone cells. This threshold corresponds to an approximate ≥4-fold difference in template abundance and was selected to ensure detection of enrichment clearly exceeding background signal and typical technical variability of RT-qPCR assays. This approach is consistent with commonly adopted fold-change-based thresholds in enrichment assays; however, it should not be considered fully quantitative, but rather a method to evaluate binary transfer of the material.

### 2.12. Animals

All animal experiments were conducted in accordance with applicable national and international regulations governing the use and care of laboratory animals. Procedures complied with the European Economic Community Council Directive 86/609 (OJ L 358, 12 December 1987), the Italian Legislative Decree 4 March 2014, n. 26 (Gazzetta Ufficiale della Repubblica Italiana no. 61), the National Institutes of Health Guide for the Care and Use of Laboratory Animals (NIH Publication no. 85-23), and the ARRIVE guidelines. The experimental protocol was reviewed and authorized by the Italian Ministry of Health (Approval No. 173/2016-PR).

### 2.13. Osteoblast Primary Cultures

Primary osteoblasts were obtained from calvariae of 7-day-old CD1 mice (6 animals/culture) of both genders. After removal of adherent soft tissues, calvarial bones were subjected to sequential enzymatic digestion using 1 mg/mL type IV collagenase (*Clostridium histolyticum*) combined with 0.25% trypsin. Three digestion steps were performed at 37 °C under gentle agitation, lasting 15, 30, and 45 min, respectively. Cells were collected from the second and third digestion fractions, centrifuged at 300 *g* for 7 min, and resuspended in DMEM supplemented with 10% foetal bovine serum. Cultures were maintained at 37 °C in a humidified incubator with 5% CO_2_. On reaching confluence, cells from the second and third digestions were detached with trypsin, pooled and replated according to the specific experimental design. Osteoblast identity and culture purity were confirmed by assessing the expression of canonical osteoblastic markers, including *Alp*, *Runx2*, *Col1a1*, *Col1a2* and *Ocn*.

### 2.14. Mineralisation Assay

Primary mouse osteoblasts were cultured in mineralization medium (DMEM + 10% FBS supplemented with 10 mM β-glycerophosphate and 50 μg/mL ascorbic acid) supplemented with miRNA mimics (i.e., miR-29a and miR-29b alone and in combination) or non-targeting miRNA (NT-miR) for 14 days, changing the medium every 3–4 days. At the end, cells were fixed in 4% buffered paraformaldehyde (PFA) for 10 min and washed twice with distilled water, then the presence of mineralization nodules was detected by von Kossa staining. Briefly, fixed osteogenic cells were incubated with 5% silver nitrate and exposed to ultraviolet light for 1 h; then, cells were washed twice with distilled water, incubated with 5% sodium thiosulfate for 2 min, washed twice with distilled water, and air dried in a fume hood. Pictures of mineralization nodules (stained brown to black) were taken under an inverted phase-contrast microscope, while quantification was performed using the NIH ImageJ tool.

### 2.15. Osteoclast Primary Cultures

Bone marrow cells were isolated from femurs and tibiae of 7-day-old CD1 mice (6 animals/culture) of both genders by flushing the medullary cavity with Hank’s Balanced Salt Solution (HBSS). The cell suspension was mixed 1:1 with HBSS, layered onto Histopaque 1077, and centrifuged at 400 *g* for 30 min to separate mononuclear cells. The interphase fraction (buffy coat) was collected, washed twice in HBSS, and resuspended in DMEM supplemented with 10% foetal bovine serum (FBS). Cells were seeded at a density of 1 × 10^6^ cells/cm^2^. After 3 h, non-adherent cells were removed by DPBS washing. To promote commitment toward the osteoclast lineage, cultures were supplemented the following day with 50 ng/mL human recombinant M-CSF. After 3 days of preconditioning, medium was replaced and cells were maintained in DMEM + 10% FBS containing (i) 50 ng/mL M-CSF alone (negative control), (ii) 50 ng/mL M-CSF plus 120 ng/mL human recombinant RANKL (positive control), or (iii) M-CSF plus miRNA mimics (alone or combined) or non-targeting miRNA mimic. On day 7, cells were fixed with 4% buffered paraformaldehyde and osteoclast differentiation was evaluated by histochemical detection of tartrate-resistant acid phosphatase (TRAcP) activity following the manufacturer’s protocol.

### 2.16. In Vitro Tube Formation Assay

The tube formation assay was performed according to Kubota and colleagues [11]. Briefly, 15-well μ-slides (Ibidi GmbH, Gräfelfing, Germany) were coated with Matrigel and allowed to solidify at 37 °C for 30 min. HUVECs cells (1.5 × 10^4^/well) were then plated and treated with miRNA mimics (i.e., miR-29a and miR-29b alone and in combination) or non-targeting miRNA (NT-miR). After 16 h, tube formation was inspected under an inverted light microscope at magnification 40×. Pictures were taken, and the percentage of branching points/area was evaluated (branching index), along with the total segments of length, using an Image J extension system for angiogenesis analysis.

### 2.17. Comparative Real Time RT-PCR

RNA from mouse primary osteoblasts and bone marrow macrophages was extracted using TRIzol reagent, then 1 μg of RNA was reverse-transcribed into cDNA using the Moloney Murine Leukemia Virus (M-MuLV) recombinant reverse transcriptase and subjected to real time PCR. All reactions were carried out using a SYBR green-based master mix no-ROX. Data were analysed via dedicated software (Light Cycler 96 SW 1.1, Roche Diagnostics GmbH, Mannheim, Germany) using the ΔΔCt method, and *Gapdh* was chosen as the housekeeping gene.

### 2.18. In Silico Target Prediction Analysis

To evaluate whether the genes modulated following miR-29a-3p and miR-29b-3p mimic treatment could represent predicted direct targets, silico analyses were conducted using three independent databases: miRDB (https://mirdb.org/, accessed on 13 February 2026), TargetScan 8.0 (https://www.targetscan.org/vert_80/, accessed on 13 February 2026), and TarBase v9.0 (https://dianalab.e-ce.uth.gr/tarbasev9/interactions, accessed on 13 February 2026). From miRDB, we extracted the target score assigned to each gene, which reflects the confidence of miRNA–target interaction prediction based on a machine learning algorithm trained on high-throughput sequencing data, with higher scores indicating greater likelihood of targeting. From TargetScan 8.0, we retrieved the total context++ score, a quantitative measure of predicted repression strength (with more negative values indicating stronger predicted repression), as well as the aggregate PCT (Probability of Conserved Targeting), which estimates the evolutionary conservation of the predicted binding site. From TarBase v9.0, we collected the reported microT scores, representing high-confidence predicted or experimentally supported miRNA–target interactions, with higher values indicating stronger evidence of interaction. The osteoblast-related genes analysed in this study were individually queried, and the corresponding parameters were extracted and summarized.

### 2.19. Statistics

Statistical analyses were performed using GraphPad Prism version 9.0 (GraphPad Software, San Diego, CA, USA). Data are reported as mean ± SEM of at least 3 independent experiments. Statistical significance between two groups was assessed using one sample *t*-test or paired student’s *t*-test, as specified in the Figure legends. A *p* value ≤ 0.05 was considered statistically significant.

## 3. Results

### 3.1. RNAs Shuttled by Breast Cancer EVs Are Transferred to Osteoblasts and Osteoclast Precursors

Starting from our previous work [9], which highlighted the ability of BrCa-EVs to influence osteoblast and osteoclast homeostasis, we aimed at elucidating the molecular mechanisms involved. It is well known that EVs contain various macromolecules and, among these, miRNAs particularly stand out. These non-coding RNAs are known in the literature for their ability to regulate gene expression and our hypothesis sees them as potentially responsible for the effects on bone induced by BrCa-EVs. We first isolated EVs from MDA-MB-231 and 4T1 cells conditioned medium (CM) and confirmed their nanoparticle nature by nanosight (Supplementary Figure S1a,b). Moreover, transmission electron microscopy (TEM) confirmed the vesicular nature of the isolated EVs and their integrity (Supplementary Figure S1c,d), while western blot analysis revealed the expression of the typical markers expressed by EVs, such as CD63, CD81 and Annexin (Anxa) 2 (Supplementary Figure S1e,f).

**Figure 1:**
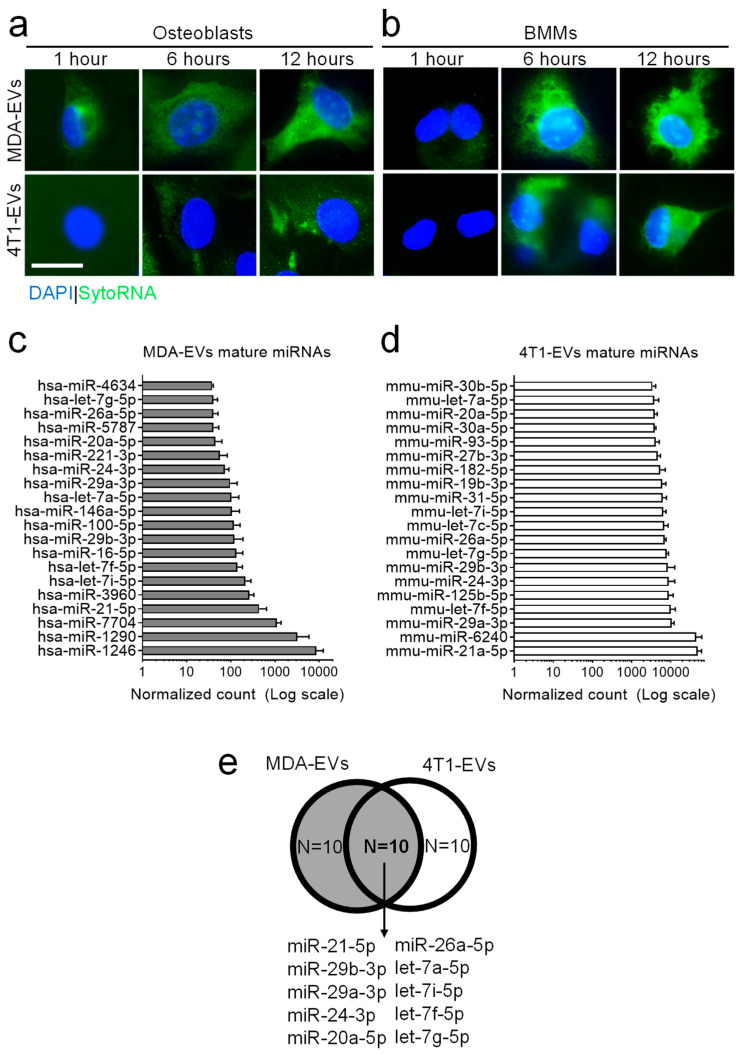
MDA-MB-231- and 4T1-EV-bound RNA transfer to bone cells. (**a**,**b**) Extracellular vesicles isolated from MDA-MB-231or 4T1 cells (i.e., MDA-MB-231-EVs and 4T1-EVs, respectively) were incubated with the membrane-permeant SYTO™ RNASelect™ probe to fluorescently label RNAs. Extracellular vesicles were added to primary mouse (**a**) osteoblasts or (**b**) bone marrow macrophages (BMMs), the latter representing osteoclast precursors. Once fixed, cells were mounted with DAPI and analysed by fluorescence microscopy. Pictures are representative of three experiments (bar = 20 μm). (**c**,**d**) List of the 20 most expressed mature miRNAs present in MDA-MB-231-EVs (MDA-EVs) and 4T1-EVs, assessed by non-coding-RNA deep-sequencing analyses. Results are from 3 independent EV preparations. (**e**) List of the 10 miRNAs shared between MDA-MB-231-EVs (MDA-EVs) and 4T1-EVs.

Next, we evaluated the ability of EVs to transfer their RNA content to target cells, i.e., osteoblasts and osteoclasts. To this end, we isolated and stained MDA-MB-231- and 4T1-EVs with an RNA-specific fluorescent probe (SYTO™ RNASelect™, Thermo Fisher Scientific) and treated both osteoblasts (Figure 1a) and osteoclast precursors, that is, bone marrow macrophages (BMMs, Figure 1b), with fluorescently tagged EVs, at different time points. Fluorescence microscopy revealed that after 1 h, despite some potential minor differences between osteoblasts and BMMs, the transfer of RNAs was low or undetectable in all conditions. A punctate intracellular staining pattern became apparent after 6 and 12 h in all cell type/EV combinations, while a weaker nuclear signal was occasionally observed. Overall, these findings indicate that RNAs associated with BrCa-derived EVs are internalised by target bone cells within 12 h of treatment.

### 3.2. miRNA Profiling of Breast Cancer-Derived EVs

Having demonstrated that RNAs contained in EVs from MDA-MB-231 and 4T1 are transferred to bone cells, we elucidated which miRNAs were most represented in these EVs. To this end, non-coding-RNA deep-sequencing analyses were conducted on three independent preparations of EVs from MDA-MB-231 and 4T1 cells. Data analysis allowed us to identify and select the 20 mature miRNAs most expressed in the EVs collected from both cell lines (Figure 1c,d). Given that, as reported in the literature [9,12], EVs from these two BrCa cells exert a similar effect on the bone microenvironment, we identified those miRNAs that were in common between the two BrCa-EVs. This allowed us to further narrow the field, identifying 10 potential candidates to analyse (Figure 1e). Finally, a literature search was carried out to exclude, among the 10 selected miRNAs, those whose role in the development of bone metastases has already been extensively investigated (Supplementary Figure S2), thus leading us to choose the following four: miR-24-3p, miR-26a-5p, miR-29a-3p and miR-29b-3p [13,14,15,16,17,18,19,20,21,22,23,24,25,26,27,28,29,30,31,32,33,34,35,36]. Moreover, since miR-21-5p has been extensively described in bone biology and metastasis [37,38,39], and it has been demonstrated to be shuttled to bone cells, we also analysed this miRNA as a positive control in our experiments.

**Figure 2:**
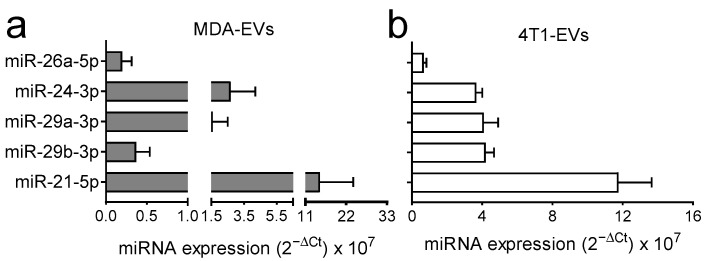
Validation of the expression of the miRNAs of interest in BrCa-EVs. Three independent RNA preparations derived from (**a**) MDA-MB-231-EVs (MDA-EVs) or (**b**) 4T1-EVs were subjected to miRNA cDNA synthesis, followed by Taqman probe-based Q-PCR to analyse expression of miR-26a-5p, miR-24-3p, mR-29a-3p, miR-29b-3p, and miR21-5p.

To confirm that the miRNAs of interest were indeed present in the EVs, we used another technique, the Taqman advanced miRNA system (Thermo Fisher scientific), and analysed the expression of the five aforementioned miRNAs. These were indeed present in MDA-MB-231- (Figure 2a) and 4T1-EVs (Figure 2b) in all the preparations analysed.

### 3.3. miR-24-3p, miR-26a-5p, miR-29a-3p and miR-29b-3p Are Transferred into Murine Osteoblasts and Bone Marrow Macrophages

**Figure 3:**
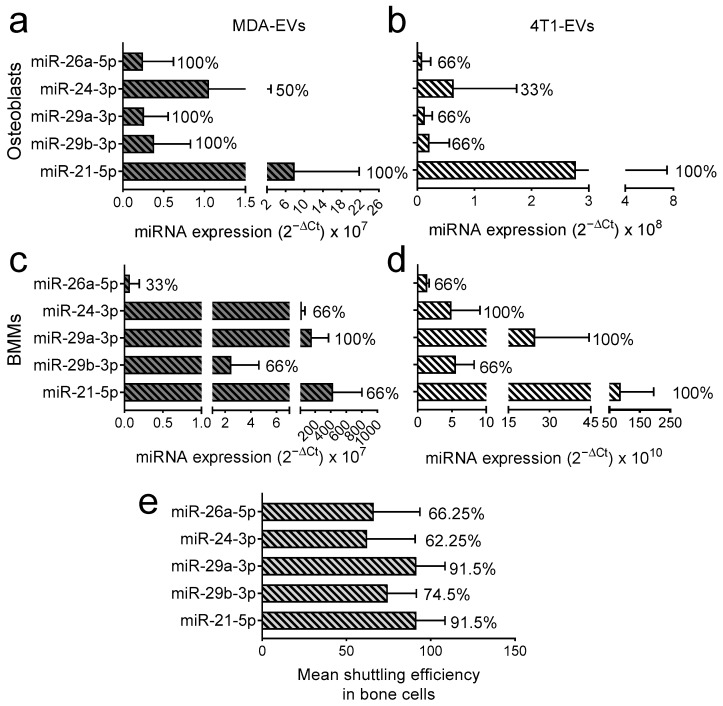
Evaluation of the efficiency of BrCa-EVs-mediated shuttling of miRNAs. MDA-MB-231 (MDA) or 4T1 cells were treated with 5-ethynyl-uridine (5-EU), a uridine analogue that is incorporated into nascent RNAs, then their EVs were isolated and used to treat (**a**,**b**) osteoblasts and (**c**,**d**) bone marrow macrophages (BMMs). After 24 h, RNA was extracted from the target cells, and the 5-EU-tagged miRNA were enriched, reverse transcribed and subjected to Q-PCR. Values written to the right side of the bars represent the % of experiments that showed detectable shuttling to bone cells. N = 3 in all panels except (**a**) where *n* = 4. (**e**) Graph summarising the efficiency of shuttling across all the conditions evaluated in (**a**–**d**).

We next investigated whether the chosen miRNAs were shuttled to bone cells via BrCa-EVs, following the flowchart described in Supplementary Figure S3 and in the Materials and Methods section (Section 2.10). Briefly, we used an affinity-tag-based approach to specifically isolate MDA-MB-231- and 4T1-EVs-derived miRNAs from target cells, which exploits 5-ethynyl-uridine (5-EU), a uridine analogue that gets incorporated into nascent RNAs. Then, we isolated the 5-EU-tagged EVs and used them to treat primary osteoblasts (Figure 3a,b) or bone marrow macrophages (BMMs), the latter being osteoclast precursors (Figure 3c,d). Then, we extracted RNAs from the target cells 24 h post-treatment and purified the 5-EU-tagged RNA molecules [40]. The presence of 5-EU miRNAs in target cells was assessed by RT-Q-PCR, as described above, and the % of experiments in which we could detect miRNA shuttling was used as the shuttling efficiency readout. As expected, miR-21-5p (positive control) showed the best overall shuttling, while miR-24-3p was shuttled with relatively low efficiency (Figure 3). In particular, for MDA-MB-231-EVs, miR-21-5p, miR-26a-5p, miR-29a-3p and miR-29b-3p were transferred into osteoblasts in 100% of the experiments conducted, while miR-24-3p was found to be transferred in only 50% of the experiments (Figure 3a). At the same time, in 4T1-EVs only miR-21-5p was transferred into osteoblasts in 100% of the experiments, while miR-26a-5p, miR-29a-3p, and miR-29b-3p transferred in 66% of the experiments and miR-24-3p in only 33% (Figure 3b). Regarding MDA-MB-231-EVs and BMMs, the transfer efficiency was 66% for miR-21-5p, miR-24-3p and miR-29b-3p; moreover miR-26a-5p was transferred in only 33% of the experiments, while only miR-29a-3p showed a transfer efficiency of 100% (Figure 3c). In the case of 4T1-EVs, however, miR-21-5p, miR-24-3p and miR-29a-3p were transferred in 100% of the experiments, while miR-26a-5p and miR-29b-3p translocated in 66% of experiments (Figure 3d).

In conclusion, we found that across all conditions, miR-29a-3p and miR-29b-3p were shuttled with efficiencies as high as 91.5% and 74.5%, respectively (Figure 3e), which made them the most promising miRNA candidates, and those which we decided to prioritize moving forward. Note that the analyses are to be considered qualitative rather than quantitative, thus providing us with a prioritized list of miRNAs with the best chance of being biologically meaningful, while confirming all of them are shuttled in at least one experiment in all recipient/EV combinations. Hence, no statistical test is presented.

### 3.4. Effect of miR-29a-3p and miR-29b-3p miRNA Mimics Treatment on Osteoblasts

**Figure 4:**
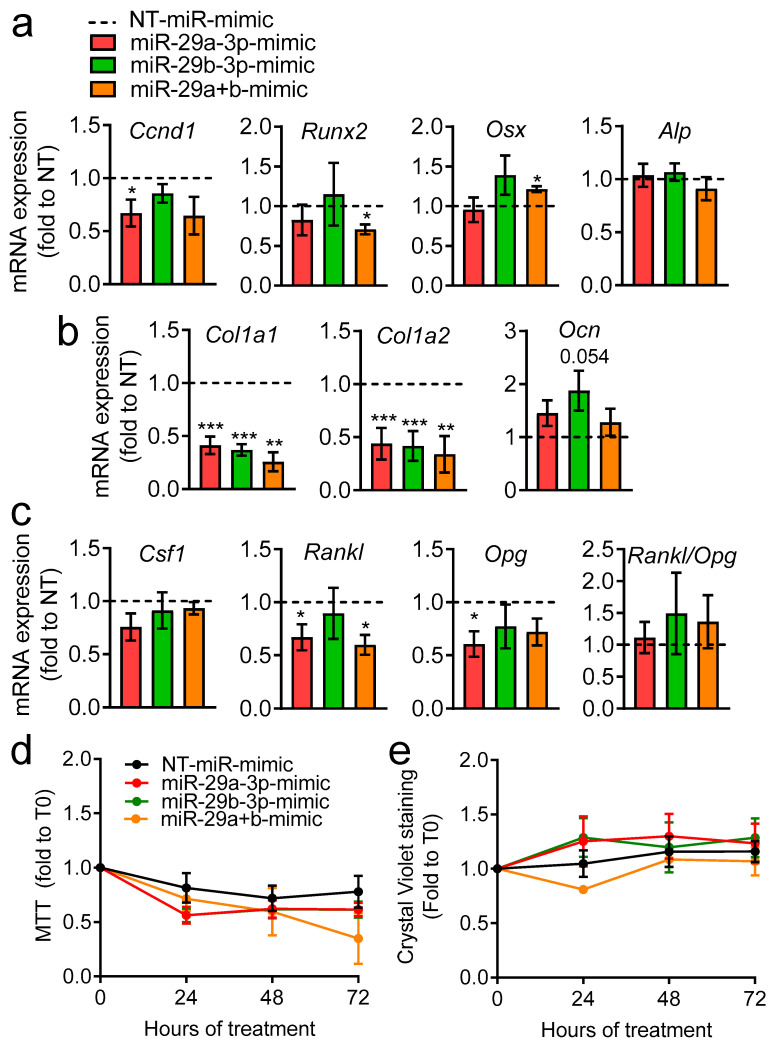
Effect of miR-29a-3p and miR-29b-3p miRNA mimics on osteoblast gene expression, viability and cell number. Mouse primary osteoblasts were treated for 48 h with 15 nM miR-29a, -29b, or a combination of miR-29a+b-mimic, and with 15 nM non-targeting (NT)-miRNA-mimic as control. Osteoblasts were subjected to RNA extraction to evaluate the expression of genes involved in osteoblast (**a**) differentiation and (**b**) bone matrix production and of (**c**) genes regulating osteoclastogenesis. (**d**,**e**) Evaluation of (**d**) metabolic activity and (**e**) cell number, assessed by MTT and crystal violet staining, respectively, in osteoblasts treated with 15 nM miRNA-mimics alone or in combination and with 15 nM NT-miRNA-mimic at the times indicated in the abscissa. Results are the mean ± SEM of 3 independent experiments (*n* = 3, * *p* < 0.05, ** *p* < 0.01 and *** *p* < 0.001 vs. non-targeting (NT)-miR-mimic), set at 1 (dotted line), one sample *t*-test).

To evaluate whether miR-29a-3p and miR-29b-3p may be mediating some of the effects BrCa-EVs cause in bone cells, we transfected primary osteoblasts with miRNA mimics for the 2 miRNAs (as a single treatment or in combination), and a non-targeting miRNA as control (NT-miR-mimic). We next performed transcriptional analysis for osteoblast differentiation genes (Figure 4a), bone matrix components (Figure 4b) and osteoblast–osteoclast coupling genes (Figure 4c). Cyclin D1 (*Ccnd1*) was reduced by miR-29a-3p-mimic, while Runt-related transcription factor 2 (*Runx2*) and osterix (*Osx*), 2 master regulators of osteoblast differentiation, were unchanged, except in the treatment with miR-29a+b, where there seemed to be a reduced expression of *Runx2*, and increased expression of *Osx* (Figure 4a). The early osteoblast differentiation marker alkaline phosphatase (*Alp*) was not affected by any of the conditions tested (Figure 4a).

Intriguingly, both miR-29a-3p and miR-29b-3p significantly reduce the expression of Collagen 1α1 (*Col1a1*) and Collagen 1α2 (*Col1a2*), which are the most abundant components in the organic bone matrix (Figure 4b). Moreover, a trend of increase (*p* = 0.054) of the late marker of osteogenic differentiation osteocalcin (*Ocn*) was found by treatment with miR-29b-3p-mimic (Figure 4b). As for osteoblast–osteoclast coupling genes (Figure 4c), we found a trend of reduction in the colony-stimulating factor 1 (*Csf1*) gene in osteoblasts treated with miR-29a (*p* = 0.09), while the receptor activator of nuclear factor κB-ligand (*Rankl*), and its decoy receptor osteoprotegerin (*Opg*), were regulated in some of the conditions tested; however, their ratio, reflecting the pro- versus anti-osteoclastogenic effect of osteoblasts, did not change (Figure 4c).

In order to explore whether the selective downregulation of *Col1a1* and *Col1a2* could reflect direct targeting by miR-29 family members, we performed in silico target prediction analyses using miRDB, TargetScan 8.0, and TarBase v9.0. MiR-29a-3p showed high-confidence prediction scores for *Col1a1* and *Col1a2* in miRDB and TarBase (Table 1). Both *Col1a1* and *Col1a2* genes were also consistently predicted as targets of miR-29b-3p across databases, displaying high miRDB target scores and strong TargetScan context++ scores, together with elevated aggregate PCT values indicative of conserved targeting (Table 2). In contrast, the other osteoblast-related genes analysed, including *Runx2*, *Osx*, *Alp* and *Ocn*, were not consistently predicted as direct targets of either miRNA across databases. These bioinformatic predictions are consistent with our in vitro results, supporting the notion that miR-29a/b primarily regulates extracellular matrix components rather than osteoblast differentiation markers.

**Figure 5:**
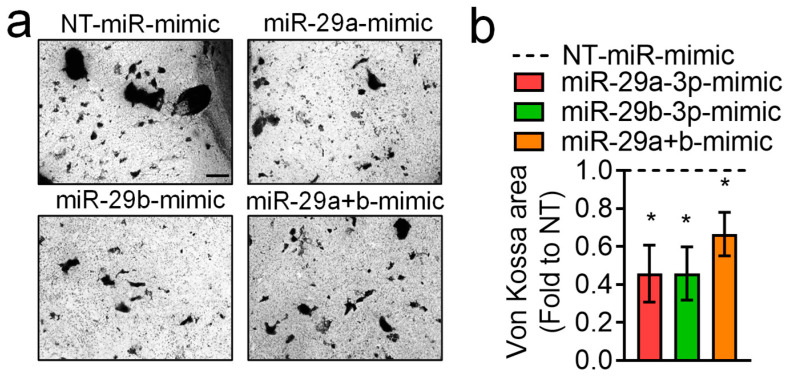
Effect of miR-29a-3p and miR-29b-3p miRNA mimics on osteoblast activity. Mouse primary osteoblasts were cultured in osteogenic medium (10 mM β-glycerophosphate and 50 μg/mL ascorbic acid in DMEM + 10% FBS) supplemented with a non-targeting (NT)-miRNA-mimic (NT-miR-mimic, 15 nM) or 15 nM miR-29a, -29b, -29a+b (combination) mimics for 14 days. Cells were then fixed in 4% PFA and subjected to (**a**) von Kossa staining (representative pictures, scale bar = 100 μm) to evaluate mineralisation nodules. (**b**) Graph showing the quantification, by Image J software, of the area of mineralised nodules, expressed as a ratio to NT control. Results are the mean ± SEM of 3 independent experiments (* *p* < 0.05 vs. non-targeting (NT)-miR-mimic, set at 1 (dotted line, one sample *t*-test).

We then evaluated cell viability via MTT assay (Figure 4d) and cell number using crystal violet staining (Figure 4e), which were unremarkable among the groups at 24, 48 and 72 h. To assess the effect of the miRNAs on osteoblasts’ mineralization ability, we evaluated mineral deposition by von Kossa staining (Figure 5), finding that it was significantly reduced in all treatments versus NT-miR, thus indicating that these miRNAs reduce osteoblast activity.

Taken together, these results show that some of the effects caused by BrCa-EVs in osteoblasts may be mediated by miR-29a-3p and miR-29b-3p.

### 3.5. Effect of miR-29a-3p and miR-29b-3p miRNA Mimics Treatment on Osteoclast Formation

**Figure 6:**
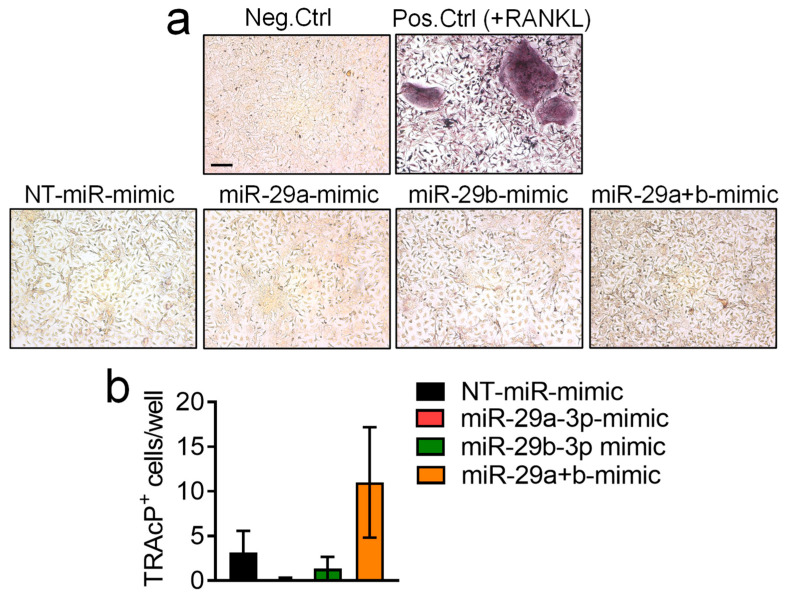
Effect of miR-29a-3p and miR-29b-3p miRNA mimics on osteoclast differentiation. Mouse macrophages, obtained from the bone marrow of 7-day-old mice (BMMs), were plated, and treated with a non-targeting (NT)-miRNA-mimic (NT-miR-mimic, 15 nM) or 15 nM miR-29a, -29b, -29a+b (combination) mimics in presence of M-CSF. Negative control (Neg. Ctrl) was done in presence of M-CSF, while positive control (RANKL) was performed by treating with 120 ng/mL RANKL plus M-CSF. After 7 days, cells were fixed in 4% PFA and subjected to (**a**) histochemical evaluation of tartrate resistant acid phosphatase (TRAcP) activity (representative pictures, scale bar = 100 μm), to (**b**) quantify the number of mononuclear osteoclast precursors and/or mature osteoclasts. Results are the mean ± SEM of 3 independent experiments. Results are not statistically significant with *p* > 0.1 in all comparisons (one-way ANOVA).

Moving to the other bone cells, that is, osteoclasts, we performed osteoclastogenesis experiments by treating murine BMMs with the pro-osteoclastogenic cytokine M-CSF and miRNA mimics, while a positive control was obtained by culturing BMMs in the presence of both M-CSF and RANKL. After 7 days, cultures were fixed and subjected to tartrate resistant acid phosphatase (TRAcP) activity histochemical evaluation to assess the number of osteoclasts (Figure 6, upper panel), and the finding was that treatment with miRNA mimics does not significantly alter osteoclastogenesis (Figure 6, graph).

### 3.6. Effect of miR-29a-3p and miR-29b-3p miRNA Mimics on In Vitro Angiogenesis

**Figure 7:**
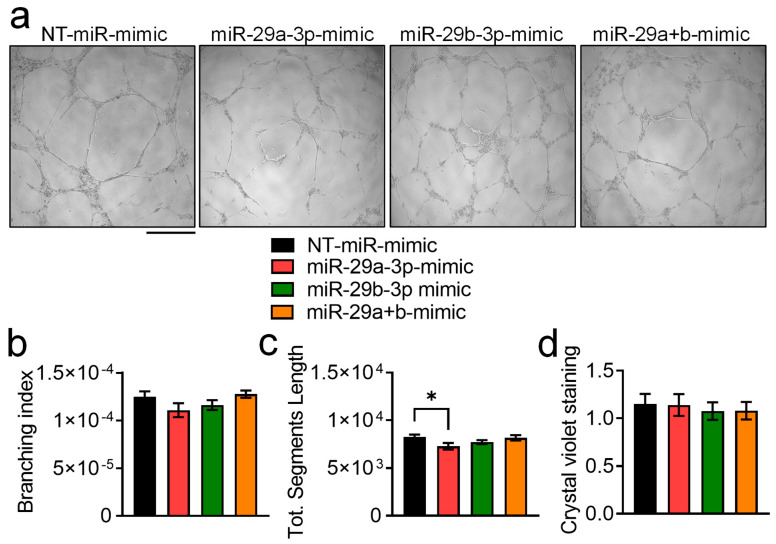
Effect of miR-29a-3p and miR-29b-3p miRNA mimics on in vitro angiogenesis. (**a**–**c**) Human umbilical vein endothelial cells (HUVECs) were treated while plated in 15-well μ-slides coated with Matrigel and treated with a non-targeting (NT)-miRNA-mimic (NT-miR-mimic, 15 nM) or 15 nM miR-29a, -29b, -29a+b (combination). After 16 h, (**a**) images were taken to evaluate (**b**) branching index and (**c**) total segments length. (**d**) Evaluation of HUVEC cell numbers by crystal violet staining. Results are the mean ± SEM of 3 independent experiments (* *p* = 0.024 vs. NT-miRNA mimic and unpaired Student’s *t*-test, scale bar = 100 μm).

Since in our previous work we observed the ability of MDA-EVs to impair in vitro angiogenesis, we finally asked whether this effect was mediated by the selected miRNAs. However, treatment with miRNA mimics seemed not to affect neither branching index (Figure 7a,b) nor endothelial cells number (Figure 7c), while only miR-29a-3p-mimic slightly reduced total segments length (Figure 7d).

## 4. Discussion

The last 15 to 20 years have been some of the most important for cancer therapy, and perhaps the most exciting advances come less from the understanding of cancer cell biology and more from the relationship cancer must establish with the microenvironment to guarantee its survival. The microenvironment is rarely a bystander in this, and often it fights back in ways that we are only starting to comprehend in both primary and secondary cancers [41].

Bone and bone marrow are quite special when it comes to microenvironment, with cellular niches changing every few microns, and with the bone matrix functioning as a store of growth factors and pro-tumorigenic molecules, which the cancer cells quickly learn to exploit through activating the so-called “vicious cycle” [42]. Although the vicious cycle can often be established by primary bone cancers, the importance of this phenomenon is more frequently reported in secondary bone cancers. Bone metastases are, in fact, the most frequent type of metastases in the most common human cancers, and we are still far from understanding exactly why this is the case [43]. What is certain is that when cancer moves to bone, it is able to throw a wrench in the wheel of bone physiology in such a way that its growth is promoted, at the expense of normal bone physiology [42]. Extracellular vesicles are an emerging local and perhaps endocrine mediator of cancer-microenvironment cross-communication. We, for example, demonstrated that BrCa-EVs exert an anti-osteoblastogenic, pro-osteoclastogenic and pro-angiogenic effect, which recapitulates well a typical breast cancer bone lesion [9]. EVs are the perfect way of transmitting complex, multi-component messages, having plenty of space for several macromolecules in their lumen, such as miRNAs, and we postulated that these could be determining some of the effects mediated by MDA-MB-231-/4T1-EVs.

There are dozens of reports showing the effects of miRNA-shuttled EVs, but it has been challenging to find a good way to demonstrate the shuttling of specific miRNAs from one cell to the other. Here, after characterizing the key miRNAs present in MDA-MB-231 and 4T1-EVs, we adapted a technique that is usually used to isolate and identify newly synthesized mRNAs and employed it to detect shuttled miRNAs in target cells instead. The technique allowed us to determine that some miRNAs are shuttled more efficiently than others (an interesting finding *per se*), and that at least the ones we prioritized also show differences when changing target cells. We can speculate that some miRNAs are quickly degraded in one cell but not so quickly in another, or even that the EV population is heterogeneous, and there is a targeted sorting of specific molecules in certain vesicles with characteristic membrane receptors, which makes them more likely to transfer pre-selected miRNAs to one cell type or the other. This could happen, for example, because of a different set of integrins expressed by the recipient cell type. This is a factor that has been demonstrated to be crucial for EVs’ organotropism [44], but there may be other mechanisms at play, including the intrinsic phagocytic tendency of recipient cells [45], which is particularly relevant when dealing with macrophages, differences in lipid rafts composition [46] and more [47]. Moving beyond the uptake, the situation becomes even more complex, with factors such as endosomal escape, miRNA stability, RISC complex readiness/saturation, mRNA abundance, and different intracellular regulatory mechanisms becoming further potential sources of differences in cellular response.

All in all, from this report, it seems that many of the vicious cycle effects are unlikely to be solely miRNA-mediated and probably involve multiple EV-associated components, which is somewhat expected given the multi-molecular nature of EVs. However, miR29a-3p and miR-29b-3p seem to be active in osteoblasts, by strongly reducing their collagen production and mineralization, which is consistent with the observed effect in vitro and in patients. Furthermore, in silico target prediction analyses performed in the present study consistently identified *Col1a1* and *Col1a2* as predicted targets of miR-29 family members (Table 1 and Table 2), which is in agreement with previous functional evidence reported in other biological contexts, including kidney [48] and foetal scleral fibroblasts [49]. Consistent with a reduction in collagen production, the analysis of nodules of mineralization in osteoblast cultures maintained for a longer time (14 days) under miRNA-mimics effect showed a significant reduction in this parameter by all three treatments (29a, 29b, 29a+b). This effect is crucial to consider in the context of bone metastases because a less dense and mineralized bone, with normal production of other factors, could be the ideal milieu for cancer growth.

It is also interesting to note a surprising counter-regulation in *Runx2* and *Osx* by the combination treatment, the former being downregulated and the latter upregulated, although both are involved in osteoblast differentiation [50]. In particular, *Runx2* is a master regulator of osteoblastogenesis [51], which is highly expressed throughout osteoblast differentiation, with a peak in preosteoblasts/immature osteoblasts, eventually decreasing in mature osteoblasts [52]. Considering this, the expression pattern would suggest that miR29a+b are delaying osteoblast differentiation. This is similar to what we observed in Loftus et al. [9], where we found osteoblast differentiation to be impacted by EVs. Regarding *Osx*, this transcription factor seems to have a role in the latter stages of osteogenesis and the maturation of mesenchymal stem cells, controlling further differentiation towards osteocytes. *Osx* is a downstream target of *Runx2* [22,24], however, the presence of an alternative regulatory mechanism independent of *Runx2* has also been identified [53]. Intriguingly, it has been recently shown that upregulation of *Osx* in the bone metastatic context is a negative prognostic factor for breast cancer bone metastases [54], suggesting that this regulation may indeed favour tumour growth in the bone milieu. The regulation of *Ccnd1* was also interesting to observe and was consistent with what MDA-MB-231-EVs cause in osteoblasts, but this regulation did not result in any meaningful difference in cell number. We can speculate that the transcriptional regulation will take more time to show its effects, or that other controllers of the cell cycle are compensating, and miR-29a is only one of the components that determine the growth repression exerted by breast cancer EVs on osteoblasts. Nonetheless, the most convincing evidence we generated here suggests that miR-29a and miR-29b are involved more intimately in controlling the matrix synthesis, rather than strongly influencing osteoblast differentiation in a radical way.

On the other hand, the role of miR29a-3p and miR-29b-3p in osteoclastogenesis and endothelial cells does not seem to be crucial, at least in a cell-autonomous manner, and the only observed difference was a small reduction in the total segment length by miR-29a-3p, but not miR-29b-3p. We could not find any reports dealing with these miRNAs in the context of osteoclastogenesis, whereas some reports are present with regards to angiogenesis, albeit results are not consistent among reports. Lamin et al. showed that ectopic expression of miR-29a in ischemic mouse hind limbs decreased angiogenesis and perfusion recovery [55], while Li et al. found that exosomal miR29a inhibited the angiogenic ability and proliferation of cardiac microvascular endothelial cell by targeting VEGF [56]. In contrast, miR29a-loaded exosomes from engineered BMSCs promote angiogenesis and osteogenesis in vivo in a non-pathological context, i.e., in 3-month-old healthy WT mice chronically and systemically for 2 months with high doses of loaded EVs [57]. With regard to miR29b, which in our hands did not affect any of the angiogenic parameters evaluated, it has been reported to have an antiangiogenic effect in hepatocellular carcinoma [58] but, for all the above, we could not find any contextualized reports relating to secondary cancers or breast cancer.

There are limitations associated with this article, including the lack of in vivo models, the still unclear relevance to human bone metastases, and the lack of selective anti-miRNA and EVs co-treatment—all valid avenues for future research. Another consideration is the use of both human (MDA-MB-231) and murine (4T1) EV-producing cell lines in combination with murine primary bone cells. Although this cross-species design may represent a limitation, the miRNAs prioritized in this study (miR-29a-3p and miR-29b-3p) are highly conserved between human and mouse, supporting their functional relevance across species. Moreover, the similarity between transfer profiles in MDA-MB-231 and 4T1 further suggests that the basic mechanisms could be similar across species. Using murine primary cells also provides a direct link to our previous work, thus allowing us to generalise our conclusions on the effects we previously observed. Nevertheless, we fully recognise that confirming these results in human-to-human experiments and within in vivo models would be the logical next step to understanding the targetability and transability of our observations. Moreover, although it is the best in relative terms, the use of miR-21 as a positive control in the shuttling experiments has its own limitations because it has not been validated directly in a similar way as we are presenting here. We infer that it is transferred, by recognising its effects are present in recipient cells, rather than directly observing its presence in recipient cells. In fact, in one of the experiments, we could not detect it in BMMs after treatment. Nonetheless, other miRNAs were detected with confidence in the same experiment, thus giving us confirmation to proceed with the analysis. Although this might seem like a detail, it underlines the complexities of the regulation and transfer of miRNAs, which should be acknowledged as a limitation. It would also be relevant to evaluate whether these miRNAs are specific to breast cancer, or whether they are also shuttled by other cancers, to understand the generalisability of the findings in relation to bone metastases.

## 5. Conclusions

In conclusion, we demonstrate that specific miRNAs are efficiently transferred from breast cancer-derived EVs to primary bone cells. Among these, miR-29a-3p and miR-29b-3p partially recapitulate the anti-osteoblastogenic effects previously attributed to tumour EVs, mainly by impairing collagen production and mineralisation. These findings indicate that EV-associated miRNAs contribute to osteoblast-specific alterations within the bone microenvironment, although additional EV cargo components are likely required to account for the broader remodelling effects previously observed. In the future, it will be important to confirm the importance of these miRNAs in vivo using human primary cell lines as recipients to confirm the transability of our findings.

## Figures and Tables

**Table 1:** Bioinformatic prediction of miR-29a-3p target genes among osteoblast-related transcripts analysed in the study.

Gene(s)	miRDB (TargetScore)	TargetScan 8.0 (Total Context+++Score)	TargetScan 8.0 (Aggregate PCT)	TarBase v9.0 (Micro_Tscore)
*Col1a1*	95	-	-	0.47
*Col1a2*	78	-	-	0.92

**Table 2:** Bioinformatic prediction of miR-29b-3p target genes among osteoblast-related transcripts analyzed in the study.

Gene(s)	miRDB (TargetScore)	TargetScan 8.0 (Total Context+++Score)	TargetScan 8.0 (Aggregate PCT)	TarBase v9.0 (Micro_Tscore)
*Col1a1*	95	−1.34	>0.99	0.98
*Col1a2*	78	−0.46	0.89	0.93

## Data Availability

The original contributions presented in this study are included in the article/Supplementary Materials. Further inquiries can be directed to the corresponding author.

## Supplementary Materials

The following supporting information can be downloaded at: https://www.mdpi.com/article/10.3390/pharmaceutics18030317/s1, Figure S1: Characterization of breast cancer cells-derived EVs; Figure S2: Flow chart showing the strategy of selection of the miRNA of interest; Figure S3: Schematic workflow illustrating the strategy used to demonstrate BrCa-derived extracellular vesicle (EV)-mediated transfer of miRNAs into recipient bone cells; Table S1: List of mouse primer sequences (5′ to 3′). Primers were designed to anneal at 60 °C.

## Abbreviations

The following abbreviations are used in this manuscript:

*Alp*	Alkaline phosphatase
BMMs	Bone marrow macrophages
BrCa	Breast cancer
*Ccnd1*	Cyclin d1
*Col1a1*	Collagen 1a1
*Col1a2*	Collagen 1a2
*Csf1*	Colony stimulating factor 1
EVs	Extracellular vesicles
OBs	Osteoblasts
*Ocn*	Osteocalcin
*Opg*	Osteoprotegerin
*Osx*	Osterix
*Rankl*	Receptor Activator of Nuclear Factor (kappa) B Ligand
*Runx2*	Runt-related transcription factor 2
TRAcP	Tartrate Resistant Acid Phosphatase
