# Supplementary material for: Extracellular Vesicles from Osteotropic Triple-Negative Breast Cancer Cells Transfer miRNAs to Bone Cells Reducing Collagen Expression and Bone Matrix Mineralisation
Source: Pharmaceutics. 2026 Mar 2;18(3):317. doi: 10.3390/pharmaceutics18030317 (PMC13029622; doi:10.3390/pharmaceutics18030317)
Supplement: Supplementary file 1 [file pharmaceutics-18-00317-s001.zip › pharmaceutics-4095737-supplementary.pdf]

pharmaceutics

MDPI

Supplementary Materials: Extracellular Vesicles from Osteotropic Triple-Negative Breast Cancer Cells Transfer miRNAs to Bone Cells Reducing Collagen Expression and Bone Matrix Mineralisation

Luca Giacchi, Argia Ucci, Elisa Pucci, Loreto Lancia, Fanny Pulcini, Simona Delle Monache, Nadia Rucci and Marco Ponzetti

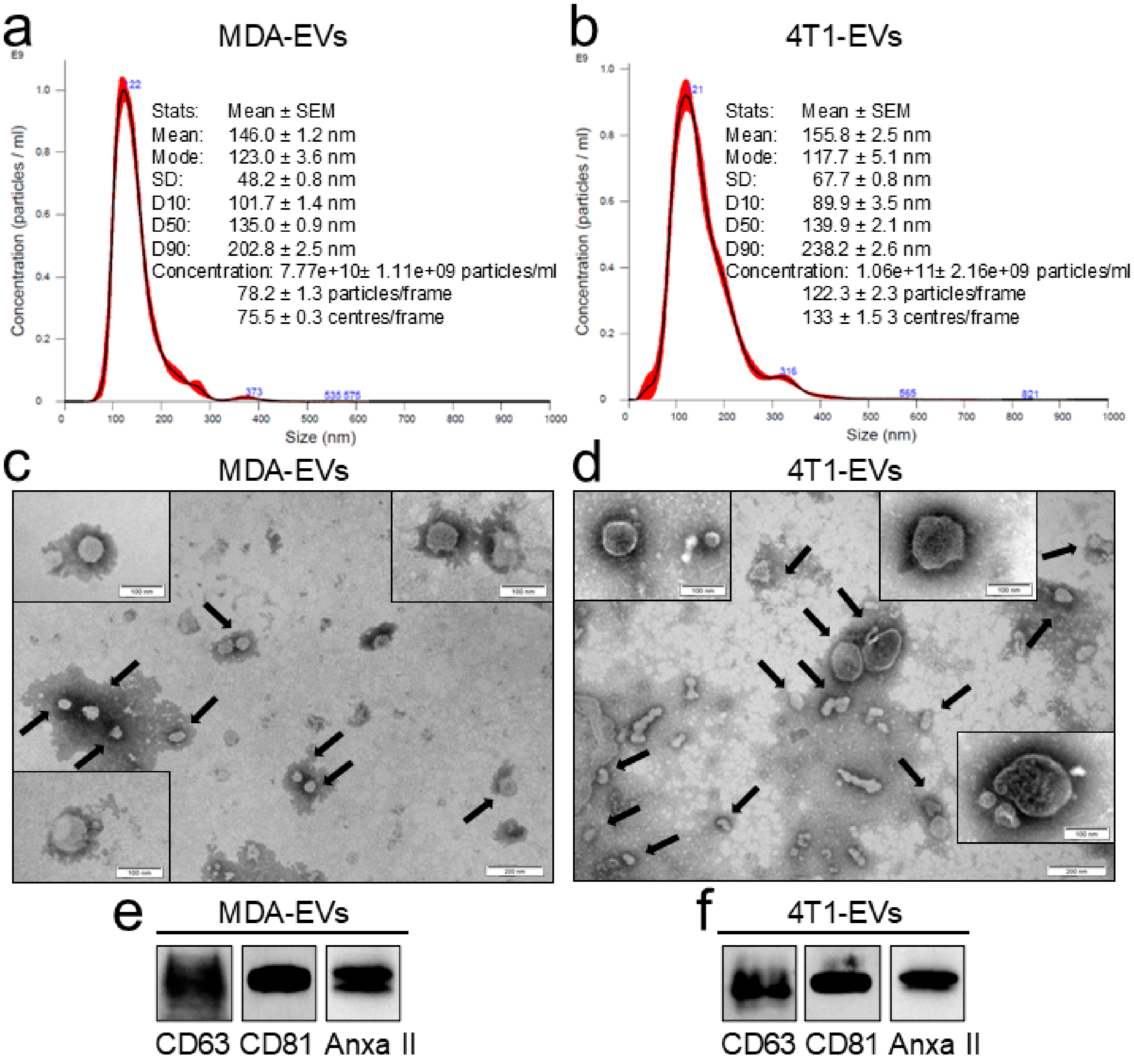

Figure S1. Characterization of breast cancer cells-derived EVs. The human and mouse breast cancer cell lines MDA-MB-231 and 4T1 were starved in serum-free DMEM for 24 hours and EVs were isolated from conditioned medium (CM) by ultracentrifugation. **(a,b)** Size and concentration evaluation of **(a)** MDA-MB-231(MDA)-EVs and **(b)** 4T1-EVs by nanoparticle tracking analysis (NanoSight NS300). Data are representative of 3 independent preparations. **(c,d)** Transmission electron microscopy (TEM) evaluation of the morphology of **(c)** MDA-EVs and **(d)** 4T1-EVs (indicated by arrows) Scale bar = 200 µm. Insets: higher magnification of the same EVs sample (scale bar = 100 µm). **(e,f)** Western blot analysis for the EVs markers CD63, CD81 and Annexin II (Anxa2, performed on proteins (15 µg loaded) extracted from **(e)** MDA-EVs and 4T1-EVs.

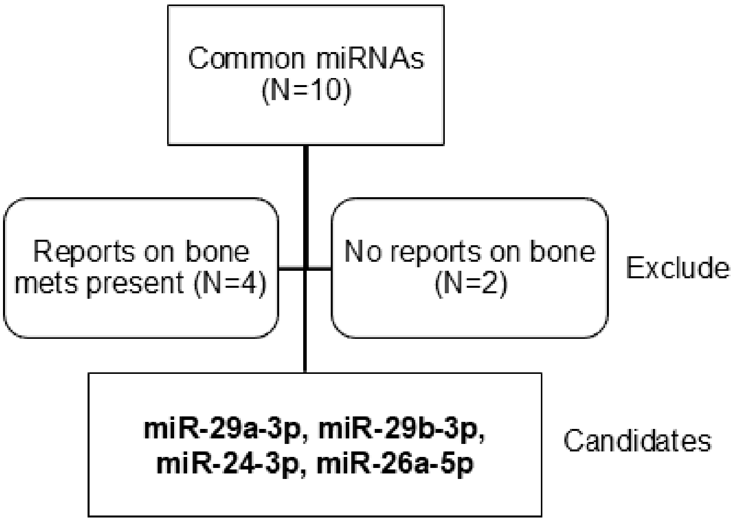

Figure S2. Flow chart showing the strategy of selection of the miRNA of interest.

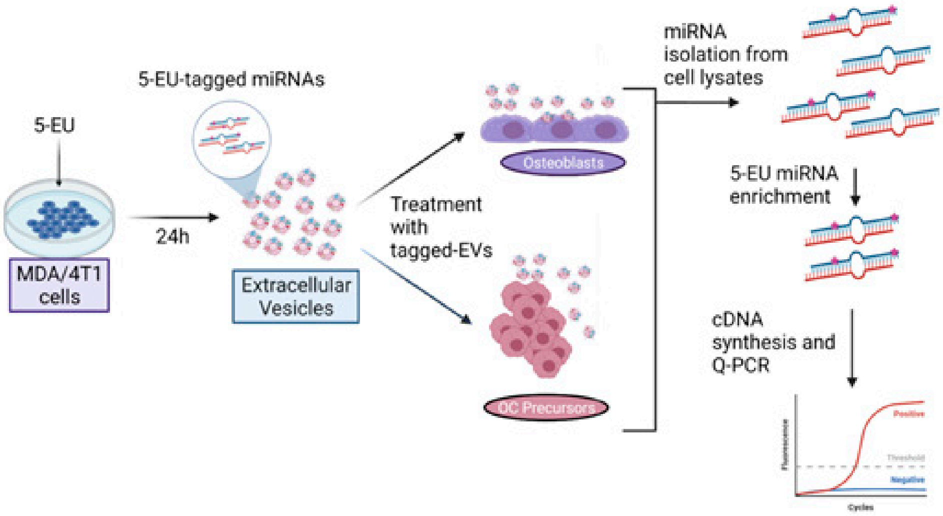

Figure S3. Schematic workflow illustrating the strategy used to demonstrate BrCa-derived extracellular vesicle (EV)–mediated transfer of miRNAs into recipient bone cells. MDA-MB-231 and 4T1 cells were metabolically labelled with 5-ethynyl uridine (5-EU) for 24 h to tag newly synthesized RNAs, and EVs were subsequently isolated and used to treat osteoblasts and osteoclast precursors. miRNA extracted from recipient cells were subjected to click chemistry–based biotinylation to selectively enrich 5-EU–labelled miRNAs, followed by streptavidin bead capture, cDNA synthesis, and RT-PCR analysis.

Table S1. List of mouse primer sequences (5′ to 3′). Primers were designed to anneal at 60 °C.

Gene ID	Forward primer	Reverse primer
Gapdh	TGGCAAGTGAGGATTGTTGC	AAGATGGTGTAGGGCTTCCCG
Alp	CCAGCAGGTTCTCTCTTGG	CTGGGAGTCTCATCCTGAGC
Runx2	AACCCACGGCCCTCCCGTGAACTCT	ACTGGCGGGGTGTAGGTAAAGGTG
Rankl	CCAAGATCTCTAACATGACG	CACCATCAGCTGAAGATAGT
Opg	AAAGCACCCTGTAGAAAACA	CCGTTTTATCCTCTCTACACTC
Osx	TGCTTCCCAATCCTATTTGC	AGAATCCCTTTCCTCTCCA
Col1a1	GTCCCTCTGGAAATGCTGGAC	GACCGGGAAGACCGACCA
Col1a2	CCGTGCTTCTCAGAACATCA	GAGCAGCCATCGACTAGGAC
m-csf	GCTCCTGCCTACCAAGACTG	GCTGTCCCACCCTTTGAATA
Ocn	TTCTGCTCACTCTGCTGACC	GGGACTGAGGCTCCAAGGTA
Cyclin D1	TCAAGTGTGACCCGGACTG	ATGTCCACATCTCGCCAGTC
